# Shiga toxin (*stx*) encoding genes in sheep and goats reared in Trinidad and Tobago

**DOI:** 10.1371/journal.pone.0277564

**Published:** 2022-11-15

**Authors:** Anil K. Persad, Gireesh Rajashekara, Jeffrey T. LeJeune

**Affiliations:** 1 Center for Food Animal Health, Ohio Agriculture Research and Development Center, The Ohio State University, Wooster, Ohio, United State of America; 2 School of Veterinary Medicine, Faculty of Medical Sciences, The University of the West Indies, Eric Williams Medical Sciences Complex, Mt. Hope, Trinidad and Tobago, West Indies; West Bengal University of Animal and Fishery Sciences, INDIA

## Abstract

Shiga toxin-producing *Escherichia coli* (STEC) is estimated to cause over two million cases of human disease annually. Trinidad and Tobago is one of the largest livestock producer and consumer of sheep and goat meat in the Caribbean, however, the potential role of these animals in the epidemiology of STEC infections has not been previously described. To fill this critical gap in knowledge, the prevalence of Shiga toxin genes *(stx*_*1*_ and *stx*_*2*_*)* shed in the faeces of healthy sheep (n = 204) and goats (n = 105) in Trinidad was investigated. Based on PCR screening, goats had a higher *stx* prevalence than sheep (46% vs 35%, *P* = 0.06). Most of the recovered STEC isolates were positive for *stx*_*1*_ only; and only three isolates were positive for the *eae* gene. None of the recovered isolates belonged to the O157 serogroup. In both species, the prevalence of *stx* was higher in young animals versus older animals. Sheep reared on deep litter flooring (43%) had a higher prevalence than sheep reared other flooring types, however this was not the same for goats. The presence of cows on the same premise was not an associated predictor for STEC carriage in sheep or goats. This study demonstrates that although sheep and goats in Trinidad are reservoirs for *stx*-positive *E*. *coli* isolates, no fecal samples tested positive for O157 STEC, harbored. Furthermore, it appears that non-O157 *stx*-positive isolates harbored by these animals do not pose a significant threat to human health

## Introduction

The annual foodborne disease burden in Trinidad and Tobago is estimated to be over 100,000 cases (almost one tenth of the population), but the true incidence and etiology of most cases is unknown since most cases are unreported [1]. Globally, STEC, a diverse group of bacteria with over 400 serogroups, is estimated to cause over two million cases of human disease annually worldwide [2]. Historically, STEC O157:H7 has been the most reported pathotype linked to human disease, however due to improved awareness and diagnostics capabilities, the number of human cases associated with non–O157 serogroups has been increasingly recognized [3–6]. Humans are primarily infected via consumption of contaminated food or water, but they can also be infected via direct contact with animals and/or their environment and via person to person transmission [7]. Infections in humans can be asymptomatic, or present with a spectrum of clinical signs including mild to bloody diarrhea. Most infections are self-limiting, but approximately 5% of cases progress to hemorrhagic uremic syndrome, thrombotic thrombocytopenic purpura and possibly death. Elderly persons, young children and immuno-compromised persons are at greatest risk of developing these complications [6, 7].

Shiga toxin-producing *Escherichia coli* (STEC) can be harbored within the gastrointestinal tract of many animals and ruminants, including sheep and goats. These small ruminants have also been identified as potential reservoirs of STEC O157:H7 and non-O157 serogroups in other geographical regions, but limited data are available for the Latin America and the Caribbean [8, 9]. The role of small ruminants in the epidemiology of STEC is exemplified by sheep being identified as the STEC reservoir of significance in Australia [7]. Similar to their bovine counterpart, these small ruminants tend to be asymptomatic shedders of STEC [10, 11]. Sheep and goats have been cited as reservoirs for over 100 other serotypes of STEC including O26, O115, O128 and O130 [12, 13]. The predominant STEC serotype shed may vary with geographic location and *stx* prevalence in animals may vary due to a number of factors including age of animals, diet, climate and sanitation [7, 9, 14]. For example, in Switzerland the main STEC serotypes shed by sheep are O91:H- and O128:H2 while the main serotypes harbored by sheep in Brazil are O76:H19 and O65:H–[15, 16]. The influence of diet on sheep STEC O157 prevalence was described by Fraser et al. (2013) who reported sheep fed brassica diets were found to have lower prevalence than animals fed conventional forage diets [14], while other authors have reported that abruptly switching sheep to low quality forages increases the shedding of STEC O157 [17]. Although O157 shedding has been demonstrated to be seasonal, with higher prevalence during warmer periods compared to colder temperatures [14, 18], no such shedding pattern has been reported for non-O157 STEC with prevalence rates remaining almost constant irrespective of season [19, 20].

Human infections have also been linked to the consumption of lamb, mutton and goat meat [21, 22]as well as consumption of unpasteurized milk and cheese made from contaminated caprine and ovine milk [11, 23]. The risk of transmission of STEC from small ruminants to humans is demonstrated by Brandal et al (2012)., who reported that almost 50% of the sheep STEC O26 isolates recovered in Norway had similar MLVA profiles to that found in human clinical cases [12]. Schimmer et al. (2008) also identified human STEC O103:H25 infections in Norway as having originated in sheep products [24]. Other reports have also implicated sheep as potential sources of human infection [13, 25, 26]. Many human infections are attributed to contact with sheep and goats at petting zoos and open farms [27, 28]. Small ruminants, especially goats, generally exhibit inquisitive behavior, thus may have greater contact with humans increasing the potential for transmission [13]. Environmental contamination with STEC shed by sheep could also be a source of human infection, as was demonstrated in Scotland when 20 scouts were infected with *E*.*coli* O157 after camping on lands which were previously grazed by sheep [29].

Sheep and goat meat is considered a delicacy in Caribbean islands. Trinidad and Tobago, located in the southern Caribbean, is one of the leading consumers of sheep and goat meat per capita in the world with over 3 kg of lamb and goat meat consumed annually per capita [30, 31]. Livestock husbandry practices in Trinidad mirrors that of the other Caribbean islands; most farms are semi-intensive and it is not uncommon to find more than one ruminant species on a farm. Most sheep and goat are primarily reared for meat production. In fact, Trinidad and Tobago is also one of the largest livestock producers in the Caribbean with an estimated 40,000 ruminants reared including 7,615 goats and 13,388 sheep [32]. Animals are frequently slaughtered under low-input, non-commercial, backyard or roadside locations in crudely constructed facilities usually devoid of running water and proper sanitation protocols. The high consumption of sheep and goat meat, coupled with poor slaughter practices, increases the risk of persons in Trinidad and Tobago contracting foodborne STEC infections from these animals.

Although being a significant cause of foodborne disease globally, there is a limited published literature on STEC epidemiology in Trinidad and Tobago. Literature searches of computer indexed databases yielded no recent publications describing the epidemiology of STEC carriage in live sheep and goats reared in this part of the world. Our literature search did however yield two publications from the early 1990s, with one study comparing the STEC prevalence in diarrheic and non-diarrheic sheep [33] and another reporting the prevalence in goat meat sold at market [34]. Neither study reported the serotype nor the virulence profile of the recovered STEC isolates.

While there are defined methods for the isolation of STEC O157, this is not the case for non-O157 serotypes [35]. For this study we used detection of Shiga toxin genes (*stx*), the main virulence factor of STEC, as an indicator for STEC presence. *Stx* is rarely reported in micro-organisms other than *E*. *coli*. The prototypic *Stx*-encoding organism is *Shigella spp*. but ruminants are not susceptible to colonization by *Shigella spp*. [36]. Given the critical void in information on STEC epidemiology in Trinidad and the wider Latin America and Caribbean, we sought to partially fill this gap in knowledge by (1) determining prevalence of *stx* in the feces of sheep and goats, (2) identify any management risk factors, and (3) evaluate the virulence profile of STEC strains recovered.

## Material and methods

A cross sectional study was carried out to determine the prevalence of *stx* in healthy sheep and goats during January to March 2015. A total of 204 sheep and 105 goat faecal samples were collected from 10 sheep and 7 goat farms in northern and central Trinidad. No data were available for the overall number of sheep and goat farms in Trinidad and their herd sizes. Farms were selected, based primarily on the farmers’ willingness to participate in the study. At each farm visit we collected a minimum of 20 sheep faecal samples and/or 10 goat faecal samples. Using previously published prevalence estimate data for sheep and goats this sampling number per farm would have given us a greater than 95% probability of detecting a STEC positive animals on the farm [33, 37, 38]. Animals with a recent history of diarrhea, other illness, or receiving antibiotics were excluded from this study. Selected farm management practices were assessed using a questionnaire and discussion with the farmers during farm visits. The key areas of focus were age of animals, housing, management system, and proximity to cattle.

De–identified human diarrheal fecal samples were kindly provided by the North-Central Regional Health Authority Adult Hospital, Mt. Hope, Trinidad and Tobago. No Epidemiological information was provided for the human fecal samples. The need for consent was waived by the ethics committee. All tests were completed in compliance with institutional (OSU) guidelines for the Care and Use of Animals and human subjects and the Ethics Committee of the Faculty of Medical Science, The University of the West Indies, St, Augustine

Collection of small ruminant faecal samples: Faecal samples were collected from sheep and goats housed on ten sheep and seven goat farms. During each visit, individual faecal samples were collected aseptically per rectum from these small ruminants using sterile gloves. These samples were placed into individually labeled Whirl-pak® bags (Nasco, Fort Atkinson, WI) and transported on ice in a cooler to the laboratory and processed within 24 hours of collection.

Processing of faecal samples: At the laboratory, faecal samples were screened for the presence of *stx* genes using a method adapted from LeJeune et al (2006) and Hu et al (1999) [36, 39]. Briefly, individual faecal samples were crushed and mixed to attain a homogenized sample. A five-gram aliquot was inoculated into 45 milliliters of Buffered Peptone Water (BPW) (Acumedia, East Lansing, MI). This suspension was then thoroughly mixed via manual agitation for two minutes and incubated overnight at 42°C. Following enrichment, 100 μl was removed for detection of Shiga toxin genes (*stx*_1_ and *stx*_2_) and 200 μl of the enrichment mixed with Buffered Glycerol and frozen at -80°C for further analysis at a later date.

Screening for *stx* genes: 100 μl of the BPW enrichment was added to a 1.7ml microcentrifuge tube containing 900μl of BPW. The contents of the tube were then mixed using a vortex and the cells pelleted via centrifugation at 18,000 x g for three minutes. The supernatant was decanted and pellet resuspended in 1 ml of BPW. This procedure was repeated three times. After the third washing procedure, the enriched faecal suspensions were boiled at 100°C for 15 minutes. The suspensions were then allowed to cool to room temperature and 4 μl RNAse (0.5 mg/ml) was added to each sample and incubated for 30 minutes at 37°C. The cell lysates were then used as the DNA template for PCR screening for the detection of *stx*_*1*_ (210 bp) *and stx*_*2*_ (484 bp) genes as described by Hu et al (1999) [39]. Amplifications were performed in a 100μl reaction mixture containing 50μl GoTaq® Green Master Mix (Promega, Madison, WI), 3.0 μl of each primer, SLT IF (5’ TGTAA CTGGAAAGGTGGAGTATAC3’), SLT IR (5’GCTATTCTGAGTCAACGAAAAATA AC3’), SLT IIF (5’GTTTTTCTTCGGTATCCTATTCCG3’), SLT IIR (5’GATGCATC TCTGGTCATTGTATTAC3’), 2.5 μL MgCL_2_, 30.5 μl sterile water and 5μl DNA template. The reaction mixture was heated to 94°C for three minutes, followed by 30 cycles of amplification at of 94°C for 30 seconds, 59°C for one minute, one minute at 72°C, and final extension was done at 72°C for 10 minutes. The PCR products were separated on 2% agarose gels stained with GelRed (Biotium, Cambridge Bioscience Ltd, UK) and visualized using UV light. Samples testing positive for *stx* genes were subjected to further testing to identify the *stx* profile and virulence genes of individual colonies.

Screening of individual colonies: 100μl of each of the previously frozen enriched small ruminant faecal samples, were added to 1.7ml microcentrifuge tubes containing one milliliter of BPW and incubated for 18–24 hours at 42°C. Following incubation, 75μl of this enrichment was then streaked for colony isolation onto MacConkey (MAC; Acumedia, East Lansing, MI) agar plates and incubated for 18–24 hours at 42°C. The next day, up to 30 individual lactose-fermenting, suspected *E*. *coli* colonies were selected from the MAC plates. These colonies were 1) transferred to Whatman FTA Cards (GE Healthcare, Piscataway, NJ) and the cards shipped to Food Animal Health Program (FAHRP), OARDC in Wooster, Ohio for *stx* screening; and 2) inoculated into Brain-Heart infusion broth (BHI, Acumedia, East Lansing, MI) in 96-well plates. These 96-well plates were incubated for 18–24 hours at 42°C. Subsequently buffered glycerol added to each well and stored at -80°C.

At the FAHRP laboratory, a 6mm sterilized paper punch was used to excise portions of the FTA card that contained the colony smears. These portions of the card were transferred to 1.7ml microcentrifuge tubes containing 400μl of Millipore water. Tubes were vortexed three times for twenty seconds to dislodge attached cellular debris. The rinsate was then aspirated and 150μl of Millipore water added to each microcentrifuge tube. DNA elution was completed by placing the tubes in a water bath at 95°C for thirty minutes. The DNA extract was transferred to clean microcentrifuge tubes and stored at -20°C. Following DNA extraction, 10μl of DNA extract from 5 colonies were pooled together and subjected to pooled PCR screening for detection of *stx* genes as described previously. The PCR products were separated on 2% agarose gels stained with ethidium bromide and visualized using UV light. From positive pools, the individual colonies comprising each pool were also subjected to PCR screening using the same amplification conditions as stated above. Extracted DNA from *stx-*positive colonies were screened for the presence of *eae* (368 bp), *rfb* (292 bp), and *fliC* (625 bp)genes described by Hu et al (1999) [39]. Amplifications were performed in a 50μl reaction mixture containing 25μl GoTaq® Green Master Mix (Promega, Madison, WI), 1.0 μl of each of forward and reverse primers for *fliC (*F:*5’*GACTGTCGATGCATCAGGCAAAG3’; R:5’CAACGGTG ACTTATCGCCATTCC3’) and *eae* (F:5’GACTGTCGATGCATCAG GCAAAG3’; R: 5’TGGAGTATTAACATTAACCCCAGG3’) and 1.5 μl of forward and reverse primers from *rfb (F*:*5’*GTGTCCATTTATACGGACATCCATG3’; R:5’ CCTATAACGTCATG CCAATATTGCC3’), 2.5μL MgCL_2_, 0.5 μl BSA, 10.0 μl sterile water and 5μl DNA template. The reaction mixture was heated to 94°C for three minutes, followed by 30 cycles of amplification at of 94°C for 30 seconds, 59°C for one minute, one minute at 72°C, and final extension was done at 72°C for 10 minutes. The PCR products were separated on 2% agarose gels stained with ethidium bromide and visualized using UV light.

### Statistical analysis

Animal and herd prevalence values for *stx* were calculated. Statistical analyses were done using Minitab 16.0® statistical software (Minitab Inc., State College, Pa., USA.). Univariate differences in the frequency of specific on farm management practices were compared using Fisher Exact Test. Multivariable backward stepwise regression analysis was used to identify potential risk factors for the presence of one or more stx-positive sheep or goat on Trinidad farms. Potential predictor variables were assessed for collinearity using Spearman rank correlation. If the correlation value between two independent values was greater than |0.7| (*P*<0.05) then, based on biological plausibility, only one of the variables would be selected for inclusion in model [40] Differences were considered to be statistically significant at *P* <0.05.

## Results

We tested 204 sheep, 105 goat and 45 human faecal samples. We classified animals less than three months as being young (milk and grain-based diet) and those greater than three months as being adult (forage-based diet). Animals were housed either under fully intensive (zero grazed) or semi-intensive production systems. Those under semi-intensive productions systems were usually allowed to graze during the day and confined to pens at night. Due to the risk of praedial larceny, most farms we visited confined the goats to pens and were not allowed to graze. The diets of all animals were supplemented with commercially produced pellet rations. All farms provided chlorinated water for drinking to the animals. Pen flooring differed between farms, with animals being reared on either wooden slatted flooring, solid concrete, or on deep litter. Cattle were also reared on some of these farms and we investigated this as a possible risk factor for *stx* in sheep and goats.

Overall, the *stx* prevalence in goat faeces (45%, 48/105) was higher than that of sheep (35%, 71/204; *P* = 0.06). All sheep farms (100%) and six of seven goat farms (88%) sampled had at least one animal shedding *stx* in its feces. On farms which reared both sheep and goats, the prevalence in goats was higher than sheep (48% vs 32%; *P* = 0.029)

When stratified according to age, the *stx* prevalence in faecal samples from young goats was higher compared to adult goats (56% vs 39%; *P* = 0.11). The *stx* prevalence in goats which were allowed to graze was slightly lower than goats that were allowed to graze intermittently (45% vs 46%; *P* = 0.99). Goats which were reared on farms with cows had a higher *stx* prevalence compared to goats reared on farms without cows (50% vs 43%; *P* = 0.55). The *stx* prevalence in goats reared on slatted floors was higher than goats reared on deep litter bedding (52% (95% CI: 40–64%) vs 34% (95% CI: 19–49%); *P* = 0.10).

The average within-farm prevalence for *stx* in sheep faecal samples 35%. When stratified according to age, young sheep had higher *stx* prevalence than adult sheep (55% vs 30%; *P* = 0.004). Unlike goats, sheep that were confined to pens had a higher prevalence compared to animals that were allowed to graze (36% vs 32%; *P* = 0.62). The prevalence of *stx* in sheep reared on farms with cows was 52% compared to farms without cows (27% CI *P* = 0.007). With Bonferroni’s correction, pairwise analyses for flooring type demonstrated that animals housed on deep litter had a higher *stx* prevalence of 43% (*P* = 0.007) compared to animals on slatted flooring (27%) and animals on solid concrete flooring (22%).

Based on PCR screening most of the sheep and goat faecal samples contained isolates which harbored the *stx*_1_ Shiga-toxin subtype alone (68% vs 69%, *P* = 0.90). A higher proportion of goat PCR positive faecal enrichments were *stx*_2_ positive alone compared to sheep samples (14% vs 8%; *P* = 0.55). Conversely, a higher proportion of sheep faecal samples contained both *stx*_*1*_ and *stx*_*2*_. Although PCR positive, we did not recover any *stx*-positive cells from 28% of goat samples and 20% of sheep samples (*P* = 0.37). The majority of both sheep and goat faecal samples had isolates with one *stx* profile (53% vs 60%, *P* = 0.57) while a higher proportion of sheep faecal samples had isolates with two different *stx* profiles compared to goat faecal samples (17% vs 9%; *P* = 0.27). In only 4% of goat and 9% of sheep PCR positive samples we recovered *stx*-positive isolates which were either *stx*_1_, *stx*_2_ or *stx*_1+2_-positive.

Overall, we recovered 685 *stx*-positive isolates; 453 from sheep and 232 from goats. Of this, 74% were *stx*_1_ alone, 14% were *stx*_*2*_ alone, and 12% were both *stx*_*1*_ and *stx*_2_-positive. A higher proportion of the isolates recovered from goats (79%) were stx_1_-positive alone compared to 71% for sheep (*P* = 0.035). Similarly, as *stx*_1_-positive alone isolates, a greater proportion of goat isolates were *stx*_2_-positive alone compared to sheep (18% vs 11%, *P* = 0.024). However, unlike the other two other *stx* profiles, a higher proportion of sheep isolates were *stx*_1+2_- positive compared to goat isolates (18% vs 8%; *P* <0.001). Only three *stx*-positive isolates tested positive for *eae* gene and none tested positive for *rfb*_O157_. All three *eae-*positive isolates came from sheep and were *stx*_*1*_ (alone) positive. Of these three isolates, two came from the same animal and both of these isolates were also *fliC* negative, while the isolate from the other animal was *fliC* positive. The two positive animals, one a young male and the other an adult female came from different farms; however, it should be noted that one of these farms supplies breeding stock to other farms thus the potential for dissemination of *eae*-positive isolates to other farms exists.

Risk factor analysis: Variables which were significantly associated with the presence of *stx* in sheep faecal samples were age of animals and grazing. For goats, the predictor variables included age and the flooring type. The statistically significant variables in this model, with the exception of grazing for sheep, were all associated with increased odds for the animal faecal sample testing positive. Allowing sheep to graze had a protective effect and reduced the odds of testing positive for *stx*. The odds ratio and *P* values for the significant predictors are presented in Table 1.

**Table 1:** Multivariate effects logistic regression model of associations between farm management factors and prevalence of *Stx* in sheep and goat feces.

Species	Model Predictor	Odds Risk	OR	*P*–value
		(OR)	(95% CI)	
Sheep	Age	2.89	1.28–6.54	0.01
	(Young)			
	Grazing	0.27	0.09–0.81	0.02
	Flooring	4.38	1.40–13.75	0.01
	(Deep Litter)			
Goat	Age	4.95	1.73–14.21	0.003
	(Young)			
	Flooring	8.16	1.45–45.84	0.02
	(Slatted Flooring)			

## Discussion

To our knowledge, this is the first study documenting the *stx* prevalence in goats in not only Trinidad but the Caribbean. In our study, 45% of goats and 36% of sheep tested positive for shedding STEC in their feces. However, despite this high prevalence, none of the recovered isolates belonged to the O157 serogroup and only three isolates possessed *eae* gene. The severity of diseases associated with non-O157 STEC infections is usually milder than O157 infections [41], and this coupled with the low percentage of *eae*-positive isolates indicate that *stx-*positive isolates harbored by sheep and goats in Trinidad may not pose a significant threat to human health

The prevalence of *stx* in sheep obtained in this study is higher than the one previously published report of 22% STEC prevalence in sheep reared in Trinidad (*P* = 0.05) [34]. The prevalence of *stx* in sheep and goats in this study is also higher than previously published data for other animal species in Trinidad [42–44]. One possible reason for this difference is because our method for *stx* detection is more sensitive since we included an enrichment step in our detection and recovery of *stx*-positive isolates which was absent from other Trinidad STEC studies. STEC when present in animal feces is usually present at such low numbers that it may be missed via direct plating, thus cultural enrichments are required for detectible limits to be reached [35]. Another hypothesis for the higher *stx* prevalence compared to other species is *stx-*positive serotypes found in Trinidad preferentially colonizes sheep and goats as opposed to other animal species. A similar phenomenon is seen in Australia where sheep are considered the host of significance for STEC [7]. The plausibility of this species–specificity hypothesis needs to be further investigated.

The high prevalence of non-O157 *stx*-positive isolates in both sheep and goats is consistent with previously published literature from other geographical regions [11, 26, 45, 46]. In Spain, Blanco et al. (2003) reported that 36% of lambs sampled from 63 flocks shed non-O157 STEC while the prevalence in sheep reared in New Zealand is reported at 66% [26, 47]. Other studies in Spain and Vietnam focusing on goats, reported a STEC prevalence in goats similar to what we report herein: In Spain, 48% of dairy goats sampled from 12 farms were positive for non-O157 STEC while the prevalence of STEC in goats in Vietnam was 39% [11, 46]. Within the United States, the prevalence of non-O157 STEC in goats was reported to be 14% which is lower than our study, however this U.S. study only focused on serogroups O26, O45, O103, O111 and O145 and samples were only considered positive if one of these serotypes plus *stx*_*1*_ or *stx*_*2*_ were present [37]. In our study, and others cited above, samples were classified as positive if *stx*_*1*_ and/or *stx*_*2*_ positive isolates were present and no serogroup presence criterion was included for classification samples. This difference in classification criteria may be the reason for the lower prevalence reported in the US.

The majority of faecal samples harbored *stx*-positive isolates with only one *stx* profile. Isolates with multiple *stx* profiles were recovered from 27% of the faecal samples screened. The varying *stx* profiles could indicate the animals were shedding multiple serotypes of STEC or there were serotypes with different *stx* profiles.

The preponderance of *stx*_*1*_ isolates recovered from small ruminant animals has also been recorded in other epidemiological studies [11, 15, 48–50]. This high proportion of *stx*_1_-positive isolates could be as a result of these isolates being better adapted to colonization of small ruminants than stx_2_ or stx_1+2_ -positive isolates. Another reason is that *stx*-positive isolates in small ruminants were resistant to lysogenization by *stx*_2_ bacteriophages. The exact reason for this observation needs to be further investigated.

In our study none of the *stx*-positive 685 colonies screened belonged to the O157 serogroup and only three were intimin positive. Similar results have been reported in New Zealand, where Cookson et al (2006) screened 442 *stx*-positive sheep isolates and failed to recover any O157 positive isolates. In that study, only three 0.7% (3/442) versus our 0.4% (3/685) of the *stx*-positive isolates were also *eae*-positive [47]. Shilling et al (2012) also failed to recover any O157 serogroups from 193 *stx* positive isolates obtained from sheep and goats and only 0.52% of *stx*-positive isolates screened were *eae-*positive [51]. The absence or low prevalence of O157 serogroups and *eae*-positive isolates in *stx* positive small ruminant samples was also confirmed by Cortes et al (2005) who screened 105 different STEC serotypes recovered from goat feces, and reported the serogroups neither belonged to the O157 serogroup nor were they *eae*-positive [11]. Studies which have successfully identified O157 serogroup in sheep or goat feces report a prevalence of between 1.8–11.7% [25, 52–55]. Pavez-Munoz et al (2021) also failed to recover any O157 isolates from sheep samples collected as part of a backyard production system surveillance [49]. The absence or low prevalence of O157 STEC from sheep and goat feces indicates that sheep and goats may be an unlikely source of human O157 infection.

For both sheep and goats, age of the animal was found to significantly affect the odds of an animal testing positive for *stx*. For goats, young animals were almost five times as likely to test positive as an adult goat and similarly, young sheep were three times as likely to test positive as an adult sheep. Adult animals have a more diverse gastrointestinal microbial flora than young animals and such either via competitive exclusion STEC isolates may not be able to attach and colonize to the intestinal epithelium [56]. Another possibility is that younger animals may have been colonized with different STEC serogroups which are from the adults. Other plausible explanations for this difference in prevalence include differences in diet, immune status and stress which may have made younger animals more susceptible to STEC colonization [57]. Young animals (less than 3 months) are not slaughtered in Trinidad and thus although being more likely to harbor *stx-*positive compared to adults, they would not be a food safety risk. Although they do not enter the food chain, young animals can serve as maintenance hosts and can amplify and shed *stx*-positive colonies resulting in environmental contamination and transmission to other animals. Young lamb and kids are popular at open farms and petting zoos especially with little children, and as such they can be a source of infection to humans via direct contact.

Grazing reduced odds of a sheep shedding *stx*. Animals which were allowed to graze were almost five times less likely to shed *stx* than sheep not allowed to graze. One possible reason for the higher prevalence in non-grazing animals is that they are confined to pens and there is of greater contact between animals and the feces from animals shedding *stx*-positive colonies resulting in new animals being colonized or recolonization of animals [58]. The effect of grazing as a predictor of STEC prevalence in goats could not be reliably investigated since most of the goats sampled (97/105) were reared in confined pens.

The prevalence of *stx* in sheep and goats was not affected by the presence of cows on the farm. This is consistent with research findings of Urdahl et al. (2003) who also reported that there was no difference in the distribution of *stx* in sheep reared on farms with/without cattle [58]. The fact that our study demonstrates that presence of cattle on a farm is not a risk factor for *stx* carriage in sheep and goats lends further support to the hypothesis that STEC serogroups exhibit an animal-host relationship and STEC serogroups will preferentially colonize certain animal species. Previous epidemiological studies have also postulated the existence of such a relationship since there was a high degree of disparity in the STEC serogroups recovered from different animal species reared on the same farm [58, 59]. There are over 400 different STEC serotypes and while cattle has been identified as a main reservoir for O157, it is clear there may multiple other animal reservoirs for non-O157 STEC. For example Djorodjevic et al. (2004), reported that despite cattle and sheep grazing together on the same pasture, 15 non-O157 STEC serotypes, 3 of which were also *eae*-positive were only recovered from sheep and not from cattle [57]. Accordingly, if the incidence of STEC-associated human disease is to be controlled, research should be directed towards identifying the various animal or environmental reservoirs of non-157 STEC and intervention programs tailored appropriately and not just towards cattle.

Another factor identified as a possible predictor for *stx* prevalence was the flooring type. Sheep housed on deep litter bedding were four times more likely to test positive for *stx* compared to sheep housed on slatted or solid concrete flooring. One possible reason for this is deep litter bedding allowed for greater accumulation of feces in the environment compared to solid concrete flooring which were cleaned daily and slatted flooring which allowed for feces to drop below the pen and not remain in the direct environment of the animal. The overall better pen hygiene in pens that had slatted flooring or bare concrete may account for the lower prevalence observed compared to sheep reared on deep litter. Another possibility is that although sheep do not typically consume the litter, bacterial contaminants in the litter may become aerosolized and contaminate feed and water troughs or attach to the animal hide. Additionally these aerosolized particles may become trapped in the upper airway and consequently be swallowed [60, 61]. Interestingly the opposite was observed with goats. Goats on slatted flooring were eight times as likely to test positive for *stx* compared to goats on deep litter flooring. Goats tend to prefer diets with higher roughage content compared to sheep [62], and given their browsing and inquisitive nature they are more likely to consume bedding in their pen. Consumption of high roughage bedding can result in increased undigested matter reaching the large intestine which undergoes fermentation and L- lactate is produced which is known to have antimicrobial effects against *E*. *coli* O157 and non- O157 *E*. *coli* isolates [63, 64]. Another possibility is that forage based bedding may contain phenolic compounds which can inhibit growth of *E*. *coli* and O157:H7 [65, 66]. The bedding may also harbor a diverse bacterial population and when consumed, may reduce intestinal colonization by STEC via competitive exclusion [17]. These factors may account for differences in *stx* prevalence but further research into the exact mechanism for the discrepancy in *stx* prevalence between sheep and goats is warranted.

One limitation of this study is that animals were sampled on farm and not immediately prior to slaughter when the risk of human transmission would be the greatest. Changes in diet, or stressful conditions associated with movement prior to slaughter may potentially alter the carriage of STEC. Another limitation is that not all production settings could be evaluated in this study. The effects of all potential variations in diets, housing conditions, and husbandry practices on STEC carriage could not be evaluated in this study, consequently resulting variations in the virulence profile of STEC pathotypes could have been missed. Another limitation is the isolation protocol, although our method gave a 95% probability recovering at least on stx-positive isolate, it is possible that an animal may harbor multiple stx serotypes and some of these could have been missed.

From data attained in this study, we can conclude that sheep and goats in Trinidad are important reservoirs of *stx*-positive non-O157 isolates. The absence of *eae-*positive strains in goats indicate that these strains are less pathogenic to humans since adherence of STEC to enterocytes, mainly dependent on the expression of *eae* gene, is highly correlated with pathogenicity [41]. Although the risk is low, these strains can nevertheless potentially result in disease since there are reports of *eae-*negative STEC isolates infections causing hemolytic uremic syndrome (HUS) and hemorrhagic colitis (HC) in humans [21]. Other virulence factors including STEC auto-agglutination adhesin (encoded by *saa* gene), sab, an auto-transporter associated with biofilm formation, and EibG, *E*. *coli* immunoglobulin binding protein G, have also been identified as contributing to attachment of *eae*-negative STEC isolates to the intestinal endothelial cells [67–69].

On the other hand, a low percentage (1%) of sheep faecal samples had *stx*-positive isolates which were also *eae*-positive. All of these isolates were *stx*_1_-postive alone. Isolates with *stx*_2_ or *stx*_1_ and *stx*_2_ are more likely associated with development of HUS and subsequent STEC associated complications. The risk of human disease from these *stx*_*1*_*-*positive isolates recovered from sheep though usually not as severe as *stx*_2_+*eae*-positive isolates should not be ignored; since there are case reports of severe human infections being caused by *stx*_1_-positive only STEC isolates [20]. Additionally, although there was a low prevalence of highly pathogenic STEC recovered from sheep and goats one must be cognizant of the possibility of *stx* phages from STEC isolates being lysogenized into an Enteroaggregative *Escherichia coli* strain as was the case in German 2011 O104:H4 outbreak [70].

In conclusion, we have identified age, flooring type and absence of grazing as risk factors which affect the prevalence of STEC in sheep and goats. Despite the high prevalence of *stx*-positive isolates shed by sheep and goats, STEC O157 was not recovered from any of the samples. Furthermore, the majority of isolates were *eae*-negative (99%) indicating they were unlikely to effectively colonize the human intestinal epithelium. We can therefore conclude that *stx*-positive isolates shed by sheep and goats in Trinidad may not pose a significant risk to human health.

## Supporting information

S1 Appendix (PDF)
