# Supplementary material for: Shiga toxin (stx) encoding genes in sheep and goats reared in Trinidad and Tobago
Source: PLoS One. 2022 Nov 15;17(11):e0277564. doi: 10.1371/journal.pone.0277564 (PMC9665368; doi:10.1371/journal.pone.0277564)
Supplement: S1 Appendix — (PDF) [file pone.0277564.s001.pdf]

Bank ID	Sample ID	PCR (+/-)	stx1	stx2	Bank Location
APS1	1-A-M	-			Plate A1
APS2	1-A-F	-			Plate A2
APS3	1-A-F	-			Plate A3
APS4	1-A-F	-			Plate A4
APS5	1-A-F	-			Plate A5
APS6	1-A-F	-			Plate A6
APS7	1-Y-M	+	+	+	Plate A7
APS8	1-A-F	-			Plate A8
APS9	1-A-M	+	+	-	Plate A9
APS10	1-A-F	-			Plate A10
APS11	1-Y-M	-			Plate A11
APS12	1-A-F	-			Plate A12
APS13	1-A-F	-			Plate A13
APS14	1-A-F	-			Plate A14
APS15	1-A-F	-			Plate A15
APS16	1-A-F	-			Plate A16
APS17	1-A-F	-			Plate A17
APS18	1-A-F	-			Plate A18
APS19	1-Y-M	+	+		Plate A19
APS20	1-A-F	+	+		Plate A20
APS21	1-A-F	-			Plate A21
APS22	2-Y-M	+	+		Plate A22
APS23	2-Y-M	-			Plate A23
APS24	2-Y-F	+	+		Plate A24
APS25	2-A-F	+	+		Plate A25
APS26	2-Y-M	+	+	+	Plate A26
APS27	2-Y-F	+	+		Plate A27
APS28	2-A-F	+	+		Plate A28
APS29	2-A-F	-			Plate A29
APS30	2-A-F	-			Plate A30
APS31	2-Y-F	-			Plate A31
APS32	2-Y-M	+			Plate A32
APS33	2-A-F	-			Plate A33
APS34	2-A-F	-			Plate A34
APS35	2-A-F	-			Plate A35
APS36	2-Y-M	+	+	+	Plate A36
APS37	2-Y-M	+	+		Plate A37
APS38	2-A-F	+	+	+	Plate A38
APS39	2-Y-M	-			Plate A39
APS40	2-A-F	-			Plate A40
APS41	2-Y-F	+	+		Plate A41
APS42	3-A-F	-			Plate A42
APS43	3-A-F	-			Plate A43
APS44	3-A-F	+			Plate A44
APS45	3-Y-F	-			Plate A45
APS46	3-A-F	-			Plate A46

APS47	3-A-F	-			Plate A47
APS48	3-A-M	-			Plate A48
APS49	3-A-F	+		+	Plate A49
APS50	3-A-M	+	+		Plate A50
APS51	3-A-M	+	+		Plate A51
APS52	3-A-F	+	+	+	Plate A52
APS53	3-A-F	+	+	+	Plate A53
APS54	3-Y-F	+	+	+	Plate A54
APS55	3-A-F	-			Plate A55
APS56	3-A-F	-			Plate A56
APS57	3-A-F	+		+	Plate A57
APS58	3-A-F	+	+		Plate A58
APS59	3-Y-F	-			Plate A59
APS60	3-A-F	+	+		Plate A60
APS61	3-A-F	+	+		Plate A61
APS62	3G-A-M	+		+	Plate A62
APS63	3G-A-F	+	+		Plate A63
APS64	3G-A-F	+	+		Plate A64
APS65	3G-A-F	-			Plate A65
APS66	3G-A-M	+	+	+	Plate A66
APS67	3G-A-F	-			Plate A67
APS68	3G-A-M	+	+	+	Plate A68
APS69	3G-A-F	+	+		Plate A69
APS70	3G-A-M	+	+		Plate A70
APS71	3G-A-F	-			Plate A71
APS72	4-Y-M	+	+		Plate A72
APS73	4-A-M	-			Plate A73
APS74	4-Y-F	-			Plate A74
APS75	4-A-M	+	+		Plate A75
APS76	4-Y-M	-			Plate A76
APS77	4-Y-M	+	+		Plate A77
APS78	4-Y-M	-			Plate A78
APS79	4-Y-M	-			Plate A79
APS80	4-Y-M	+	+		Plate A80
APS81	4-A-M	-			Plate A81
APS82	4-Y-M	+	+		Plate A82
APS83	4-A-F	-			Plate A83
APS84	4-Y-M	-			Plate A84
APS85	4-A-F	-			Plate A85
APS86	4-Y-M	-			Plate A86
APS87	4-A-F	-			Plate A87
APS88	4-A-F	-			Plate A88
APS89	4-A-F	-			Plate A89
APS90	4-A-F	-			Plate A90
APS91	4-A-F	-			Plate A91
APS92	4-A-F	+	+		Plate A92
APS93	4G-A-M	-			Plate A93

APS94	4G-A-M	-			Plate A94
APS95	4G-A-M	-			Plate A95
APS96	4G-Y-F	-			Plate A96
APS97	4G-Y-F	-			Plate B1
APS98	4G-Y-M	-			Plate B2
APS99	4G-Y-M	-			Plate B3
APS100	4G-Y-M	-			Plate B4
APS101	5-A-F	+	+		Plate B5
APS102	5-A-F	-			Plate B6
APS103	5-A-F	-			Plate B7
APS104	5-A-F	+	+		Plate B8
APS105	5-A-F	-			Plate B9
APS106	5-A-F	-			Plate B10
APS107	5-A-F	+	+		Plate B11
APS108	5-A-F	-			Plate B12
APS109	5-A-F	-			Plate B13
APS110	5-A-F	-			Plate B14
APS111	5-A-F	+	+		Plate B15
APS112	5-A-F	-			Plate B16
APS113	5-A-F	+	+		Plate B17
APS114	5-A-F	+	+		Plate B18
APS115	5-A-F	-			Plate B19
APS116	5-A-F	+	+		Plate B20
APS117	5-A-F	-			Plate B21
APS118	5-A-F	+	+		Plate B22
APS119	5-A-F	+	+		Plate B23
APS120	5-A-F	-			Plate B24
APS121	5G-A-F	+	+		Plate B25
APS122	5G-A-M	-			Plate B26
APS123	5G-Y-M	+	+	+	Plate B27
APS124	5G-A-F	-			Plate B28
APS125	5G-A-F	-			Plate B29
APS126	5G-A-F	-			Plate B30
APS127	5G-A-F	+	+		Plate B31
APS128	5G-A-F	-			Plate B32
APS129	5G-A-F	+			Plate B33
APS130	5G-A-F	+			Plate B34
APS131	5-A-F	-			Plate B35
APS132	6-A-F	+	+		Plate B36
APS133	6-A-F	-			Plate B37
APS134	6-A-F	-			Plate B38
APS135	6-A-F	-			Plate B39
APS136	6-A-F	-			Plate B40
APS137	6-A-F	-			Plate B41
APS138	6-A-F	+	+	+	Plate B42
APS139	6-A-F	-			Plate B43
APS140	6-A-F	-			Plate B44

APS141	6-A-F	-			Plate B45
APS142	6-A-F	+	+	+	Plate B46
APS143	6-A-F	-			Plate B47
APS144	6-A-F	-			Plate B48
APS145	6-A-F	-			Plate B49
APS146	6-A-F	-			Plate B50
APS147	6-A-F	-			Plate B51
APS148	6-A-M	-			Plate B52
APS149	6-A-F	-			Plate B53
APS150	6-A-M	+		+	Plate B54
APS151	6-A-F	+	+		Plate B55
APS152	7-A-F	+	+		Plate B56
APS153	7-A-F	-			Plate B57
APS154	7-A-F	-			Plate B58
APS155	7-A-F	+	+		Plate B59
APS156	7-A-F	-			Plate B60
APS157	7-A-F	-			Plate B61
APS158	7-A-F	-			Plate B62
APS159	7-A-F	-			Plate B63
APS160	7-A-F	-			Plate B64
APS161	7-A-F	-			Plate B65
APS162	7-A-F	-			Plate B66
APS163	7-A-F	-			Plate B67
APS164	7-A-F	+	+		Plate B68
APS165	7-A-F	-			Plate B69
APS166	7-A-F	-			Plate B70
APS167	7-A-F	+	+		Plate B71
APS168	7-A-F	-			Plate B72
APS169	7-A-F	-			Plate B73
APS170	7-A-F	-			Plate B74
APS171	7G-A-F	+	+		Plate B75
APS172	7G-A-F	+	+		Plate B76
APS173	7G-A-F	+	+	+	Plate B77
APS174	7G-A-F	-			Plate B78
APS175	7G-A-F	+	+		Plate B79
APS176	7G-A-F	+		+	Plate B80
APS177	7G-A-F	+	+		Plate B81
APS178	7G-A-F	-			Plate B82
APS179	7G-A-F	+	+		Plate B83
APS180	7G-A-F	+	+		Plate B84
APS181	8-A-F	+	+		Plate B85
APS182	8-A-F	+	+		Plate B86
APS183	8-A-F	-			Plate B87
APS184	8-A-F	-			Plate B88
APS185	8-A-F	+	+	+	Plate B89
APS186	8-A-F	-			Plate B90
APS187	8-A-F	+	+		Plate B91

APS188	8-A-F	+	+	+	Plate B92
APS189	8-A-F	+	+	+	Plate B93
APS190	8-Y-M	-			Plate B94
APS191	8-Y-M	+	+	+	Plate B95
APS192	8-Y-M	+	+	+	Plate B96
APS193	8-Y-M	+	+		Plate C1
APS194	8-Y-M	-			Plate C2
APS195	8-Y-M	-			Plate C3
APS196	8-Y-F	+	+		Plate C4
APS197	8-Y-F	+	+	+	Plate C5
APS198	8-Y-F	-			Plate C6
APS199	8-Y-F	-			Plate C7
APS200	8-Y-F	+	+	+	Plate C8
APS201	7-A-F	-			Plate C9
APS202	9-A-M	-			Plate C10
APS203	9-A-F	-			Plate C11
APS204	9-A-F	-			Plate C12
APS205	9-A-F	-			Plate C13
APS206	9-A-F	+	+	+	Plate C14
APS207	9-A-F	-			Plate C15
APS208	9-A-F	-			Plate C16
APS209	9-A-F	-			Plate C17
APS210	9-A-F	-			Plate C18
APS211	9-A-F	-			Plate C19
APS212	9-A-F	-			Plate C20
APS213	9-A-F	-			Plate C21
APS214	9-A-F	-			Plate C22
APS215	9-A-F	-			Plate C23
APS216	9-A-F	-			Plate C24
APS217	9-A-F	-			Plate C25
APS218	9G-A-F	-			Plate C26
APS219	9G-Y-F	-			Plate C27
APS220	9G-Y-M	+	+		Plate C28
APS221	9G-Y-M	+	+		Plate C29
APS222	9G-A-F	-			Plate C30
APS223	9G-Y-M	+	+	+	Plate C31
APS224	9G-Y-F	+	+		Plate C32
APS225	9G-Y-F	+		+	Plate C33
APS226	9G-A-F	+	+		Plate C34
APS227	9G-Y-M	+	+		Plate C35
APS228	9G-Y-M	+	+		Plate C36
APS229	9G-Y-F	+		+	Plate C37
APS230	9G-Y-F	+	+		Plate C38
APS231	9G-A-F	+	+		Plate C39
APS232	9G-A-F	-			Plate C40
APS233	9G-Y-F	+			Plate C41
APS234	9G-Y-F	+			Plate C42

APS235	9G-Y-M	-			Plate C43
APS236	9G-A-F	+			Plate C44
APS237	9G-A-F	-			Plate C45
APS238	9G-A-F	-			Plate C46
APS239	10-A-F	+		+	Plate C47
APS240	10-A-F	+	+		Plate C48
APS241	10-A-F	-			Plate C49
APS242	10-A-F	-			Plate C50
APS243	10-A-F	+	+		Plate C51
APS244	10-A-F	+		+	Plate C52
APS245	10-A-F	+	+		Plate C53
APS246	10-A-F	-			Plate C54
APS247	10-A-F	-			Plate C55
APS248	10-A-F	-			Plate C56
APS249	10-A-F	+	+		Plate C57
APS250	10-A-F	-			Plate C58
APS251	10-A-F	-			Plate C59
APS252	10-A-F	+	+		Plate C60
APS253	10-A-F	-			Plate C61
APS254	10-A-F	-			Plate C62
APS255	10-A-F	-			Plate C63
APS256	10-A-F	-			Plate C64
APS257	10-A-F	-			Plate C65
APS258	10-A-F	+			Plate C66
APS259	10-A-F	-			Plate C67
APS260	10-A-F	-			Plate C68
APS261	10-A-F	-			Plate C69
APS262	10-A-M	-			Plate C70
APS263	10-A-M	-			Plate C71
APS264	10G-Y-M	+			Plate C72
APS265	10G-A-F	-			Plate C73
APS266	10G-A-F	-			Plate C74
APS267	10G-A-F	-			Plate C75
APS268	10G-A-F	-			Plate C76
APS269	10G-A-F	-			Plate C77
APS270	10G-A-F	-			Plate C78
APS271	10G-A-F	-			Plate C79
APS272	10G-A-F	-			Plate C80
APS273	10G-A-F	-			Plate C81
APS274	10G-A-F	-			Plate C82
APS275	10G-A-F	-			Plate C83
APS276	10G-A-F	-			Plate C84
APS277	10G-A-M	+		+	Plate C85
APS278	10G-A-F	+	+		Plate C86
APS279	10G-A-F	+		+	Plate C87
APS280	10G-A-F	-			Plate C88
APS281	10G-A-F	-			Plate C89

APS282	10G-A-M	-			Plate C90
APS283	10G-Y-M	+	+	+	Plate C91
APS284	10G-A-M	-			Plate C92
APS285	10G-A-F	-			Plate C93
APS286	10G-A-F	-			Plate C94
APS287	10G-A-F	-			Plate C95
APS288	10G-A-F	-			Plate C96
APS289	10G-A-F	+	+	+	Plate D1
APS290	11G-A-M	-			Plate D2
APS291	11G-Y-M	+	+	+	Plate D3
APS292	11G-Y-F	+	+		Plate D4
APS293	11G-Y-F	-			Plate D5
APS294	11G-Y-M	-			Plate D6
APS295	11G-Y-M	-			Plate D7
APS296	11G-Y-M	-			Plate D8
APS297	11G-Y-M	-			Plate D9
APS298	11G-Y-F	-			Plate D10
APS299	11G-Y-F	-			Plate D11
APS300	11G-Y-F	+	+		Plate D12
APS301	11G-Y-M	+	+		Plate D13
APS302	11G-Y-M	-			Plate D14
APS303	11G-A-F	-			Plate D15
APS304	11G-Y-F	+	+		Plate D16
APS305	11G-Y-F	+		+	Plate D17
APS306	11G-Y-F	+	+		Plate D18
APS307	11G-Y-F	-			Plate D19
APS308	11G-Y-F	+	+		Plate D20
APS309	11G-Y-M	-			Plate D21
APS310		-			Plate D22
APS311		-			Plate D23
APS312		-			Plate D24
APS313		-			Plate D25
APS314		-			Plate D26
APS315		-			Plate D27
APS316		-			Plate D28
APS317		-			Plate D29
APS318		-			Plate D30
APS319		-			Plate D31
APS320		-			Plate D32
APS321		-			Plate D33
APS322		-			Plate D34
APS323		-			Plate D35
APS324		-			Plate D36
APS325		-			Plate D37
APS326		-			Plate D38
APS327		-			Plate D39
APS328		-			Plate D40

APS329		-			Plate D41
APS330		-			Plate D42
APS331		-			Plate D43
APS332		-			Plate D44
APS333		-			Plate D45
APS334		-			Plate D46
APS335		-			Plate D47
APS336		-			Plate D48
APS337		-			Plate D49
APS338		-			Plate D50
APS339		-			Plate D51
APS340		-			Plate D52
APS341		-			Plate D53
APS342		-			Plate D54
APS343		-			Plate D55
APS344		-			Plate D56
APS345		-			Plate D57
APS346		-			Plate D58
APS347		-			Plate D59
APS348		-			Plate D60
APS349		-			Plate D61
APS350		-			Plate D62
APS351		-			Plate D63
APS352		-			Plate D64
APS353		-			Plate D65
APS354		-			Plate D66
